# Assessment of Depression Severity During Coronavirus Disease 2019 Pandemic Among the Palestinian Population: A Growing Concern and an Immediate Consideration

**DOI:** 10.3389/fpsyt.2020.570065

**Published:** 2020-12-23

**Authors:** Hamzeh Al Zabadi, Thair Alhroub, Noor Yaseen, Maryam Haj-Yahya

**Affiliations:** ^1^Public Health Department, Faculty of Medicine and Health Sciences, An-Najah National University, Nablus, Palestine; ^2^Medicine Department, Faculty of Medicine and Health Sciences, An-Najah National University, Nablus, Palestine

**Keywords:** COVID-19, depression, Palestine, quarantine, lockdown

## Abstract

**Background:** Aggressive quarantine and lockdown measures were implemented as protective public health actions during the coronavirus disease 2019 (COVID-19) pandemic. Assessing the psychological effects associated with these measures is an important attempt to inform local policymakers in an early stage. Yet little is known about these effects, specifically depression, among the Palestinians. This study aimed to assess the prevalence and predictors of depression among the Palestinian community during this pandemic.

**Materials and methods:** A cross-sectional web-based survey throughout social media (Facebook and Instagram) was carried out using an anonymous online questionnaire. The validated and standardized depression, anxiety, and stress scale (DASS) was used to measure depression severity. A snowball technique recruiting the general public living in Palestine was conducted. Data were collected between 6 and 16 April 2020, which corresponded to the middle interval of strict massive lockdown in Palestine on 22 March to 5 May 2020. Multinomial logistic regression model was developed to predict depression severity.

**Results:** About 2,819 respondents filled out the questionnaire. Depression prevalence was (57.5%; *n* = 1,621). Out of them, 66% had mild/moderate severity, and 34% had severe/extremely severe degree. Depression severity was negatively associated with age {mild/moderate degree [OR (95% CI) = 0.98 (0.97–0.99)] and severe/extremely severe [OR (95% CI) = 0.96 (0.94–0.97)]} degrees compared with normal degree. Males were significantly less likely to have higher depression than females {mild/moderate degree [OR (95% CI) = 0.69 (0.57–0.85)] and severe/extremely severe [OR (95% CI) = 0.52 (0.40–0.86)]} degree. However, those who reported having inadequate food supply and lesser monthly incomes were more likely to have a higher degree of depression as compared with normal degree. Single persons were significantly more likely to have mild/moderate depression than those in a relationship [OR (95% CI) = 1.31 (1.05–1.64)].

**Conclusions:** High depression prevalence (57.5%) among the Palestinian community during the COVID-19 pandemic is a growing public health concern. It is essential to provide psychological counseling and treatment during and after the pandemic for the targeted people at high risk (young age/female gender) who were affected psychologically. Strategic long-term policy to address pandemic ramifications, including depression, by implementing comprehensive interventions taking into account socioeconomic disparities, vulnerability, and inequities, is crucial to emerge from this crisis in Palestine.

## Background

Quarantine was adopted as an obligatory means of separation and to restrict the movement of people who have potentially been exposed to a contagious disease and to limit disease spread (1). It has been used for centuries to contain infectious diseases such as cholera and the plague (2). Most recently, quarantine has been used in the coronavirus disease 2019 (COVID-19) pandemic (3).

Separation from loved ones and the loss of freedom during the quarantine are often unpleasant experiences (3), and they create dramatic psychological and emotional effects on some people, such as depression (2). Age, educational level, gender, marital status, living with other adults, having children, and other factors may play a role in psychological problem development such as depression (3). Anxiety and mood disorders are the most common mental health problems in the general population all over the world, and there are important connections between anxiety and depression and the occurrence of viral diseases (4). Therefore, the successful use of quarantine as a public health measure requires us to reduce and manage, as far as possible, the negative effects associated with it (3).

During the COVID-19 quarantine in Southwestern China, nearly 14.6% of study participants had depression, 8.3% had mild depression, 5.2% had moderate depression, and 1.1% had severe depression. The study also found that those who are single or very worried had significantly a higher level of depression than others. But those with “very good” self-perceived health and high income had lower levels of depression than others (4). Another multicenter survey involving 1,563 medical staff found the prevalence of depression to be 50.7% (5).

During the COVID-19 outbreak, Palestine had undergone massive quarantine for nearly 43 days (from 22 March to 5 May 2020). However, the psychological impact of this quarantine and its related lockdown measures among the Palestinian community is unknown. Aggressive quarantine and lockdown measures were implemented globally as precautionary and protective public health actions during the COVID-19 pandemic. Assessing the psychological effects associated with these implemented measures is an important research attempt to inform local policymakers on the evidence in an early stage. Yet no data are available regarding the level of depression among the general population in Palestine, and to the best of our knowledge, no study had evaluated this issue, specifically depression, among the Palestinian general public. This study seeks to assess prevalence and predictors of depression severity among the Palestinian community as the first important step for creating an early targeted intervention and helping people return to normal life.

## Materials and Methods

### Study Population, Sample, and Setting

The target population comprised all people who are 18 years or older and currently living in the West Bank, Gaza Strip, and East Jerusalem. We adopted a cross-sectional survey design to find the prevalence of depression among the public and to identify possible risk factors during the pandemic of COVID-19 by using an anonymous online questionnaire. A snowball sampling strategy focused on recruiting the general public living in Palestine during the pandemic was conducted. The online survey was first disseminated on Facebook and Instagram to friends, and they were encouraged to pass it on to others.

### Procedure

As the Palestinian Government recommended the public to minimize face-to-face interaction and isolate themselves at their homes, potential respondents were electronically invited. All of them completed the questionnaires in Arabic through an online survey. Expedited ethics approval was obtained from the Institutional Review Board (IRB) at An-Najah National University (Faculty of Medicine and Health Sciences). Privacy was strictly protected during the procedure. The purposes of the study and information about it were posted on the first page of the questionnaire. All respondents provided online informed consent before starting the questionnaire. The IRB approved our request for a waiver of documentation of this method of obtaining consent. Data collection took place over 10 days (6 to 16 April 2020), which corresponds to almost the middle interval of the massive quarantine in Palestine (22 March to 5 May 2020).

### Survey Development

Previous surveys on the assessment of mental health during quarantine in outbreaks were reviewed (5). The authors included additional questions related to the COVID-19 outbreak in Palestine. The structured questionnaire consisted of questions that covered several areas: (1) informed consent, (2) demographic data, (3) knowledge and concerns about the quarantine, (4) precautionary measures against the COVID-19, and (5) depression, anxiety, and stress scale (DASS) form in Arabic. DASS is an instrument that included 42 self-report items designed to measure the three related negative emotional states of depression, anxiety, and tension/stress. A short version, the DASS21, is available with seven items per scale (6). DASS showed excellent Cronbach's alpha values of 0.81, 0.89, and 0.78 for the subscales of depression, anxiety, and stress, respectively (7).

An Arabic-language version of the DASS was used. The form was adapted from a published study over the psychometric properties of an Arabic version of the DASS, in which the results supported the universality of depression, anxiety, and stress across cultures and thus provided initial support for the psychometric properties of the Arabic DASS (8).

A pilot study was performed on a small group of volunteers for feedback to identify ambiguities and difficult questions and to record the time needed to complete the online questionnaire, and therefore, very minor rewording was made to clarify some words and questions related to the COVID-19 pandemic and quarantine.

### Statistical Analysis

DASS contains three subscales, each composed of seven questions. The Depression subscale assesses dysphoria, hopelessness, devaluation of life, self-deprecation, lack of interest/involvement, anhedonia, and inertia. Subjects were asked to use 4-point severity/frequency scales to rate the extent to which they have experienced each state over the past week, as follows:

0 = Did not apply to me at all.

1 = Applied to me to some degree, or some of the time.

2 = Applied to me to a considerable degree or a good part of the time.

3 = Applied to me very much or most of the time.

Depression scores were calculated by summing the scores for the seven questions regarding depression in the DASS21 scale. The scores were between 0 and 21, and then they were multiplied by 2, so the final score for each participant was between 0 and 42. Scores and categories are shown in Table 1 (6).

**Table 1:** Depression severity original scores and the study re-adjusted scores.

**Severity**	**Depression severity original scores**
Normal	0–9
Mild	10–13
Moderate	14–20
Sever	21–27
Extremely sever	+28
**Scores in this study were re-adjusted as:**	
**Severity**	**Depression severity re-adjusted scores**
Normal	0–9
Mild/moderate	10–20
Sever/extremely sever	+21

DASS scores may be presented in five categorical levels. However, in this study, and according to standardized cutoffs, we merged mild with moderate and severe with extremely severe cutoff scores for depression (Table 1) to facilitate the multivariate analysis, as some cells showed less than five cases in some categorical independent variables, and this is usually accepted (9).

Data were entered into the 27th version of IBM SPSS (IBM SPSS Statistics for Windows, Version 27.0. Armonk, NY: IBM Corp). In this study, 2,819 individuals completed and returned the questionnaire. Descriptive analysis, median, mean, and standard deviation for continuous variables, and frequencies/percentages for categorical independent variables were conducted. Independent *t*-test was used to test for significance among continuous variables and the chi-square test for categorical variables. Variables that showed significance in the bivariate analysis (*P*-value <0.05) were included in a multinomial logistic regression model to predict the factors associated with each depression severity degree and presented as odds ratio and 95%CI.

## Results

### Characteristics of the Study Population

In this study, 2,819 individuals completed and returned the questionnaire (Table 2). The mean age of respondents was 29.47 years with SD of 10.97. We divided the population into three groups according to age: young (18–35), middle (36–53), and elderly (>53) age groups. Nearly 73.9% were in young age, and only 4% of participants were elderly. More than two thirds (72.6%) of respondents were female. Almost half of them (51.4%) were single. The majority of respondents live in the West Bank (83.5%), and only 9.6% live in Gaza. Around 55.1% (*n* = 1,552) of participants had an income of 2,000–5,000 New Israeli Shekels (555–1,388 USD) per month.

**Table 2:** Bivariate analysis of socio-demographic characteristics with depression severity (*P*-value presented was Chi-square significance; *N* = 2,819).

		**Depression severity**			
**Variables**	***N* (%)**	**Normal*n* = 1,198**	**Mild to moderate *n* =1,071**	**Severe to extremely sever*n*= 550**	***P-*value**
Age	2,819 (100)	Mean = 31.98 SD = 11.9	Mean = 28.3 SD = 10.4	Mean = 26.28 SD = 8.5	<0.001^*^ (ANOVA-test)
**Sex**					
Male	768 (27.2)	389 (32.5)	258 (24.1)	121 (22)	<0.001^*^
Female	2,051 (72.8)	809 (67.5)	813 (75.9)	429 (78)	
**Social status**					
Single	1,449 (51.4)	513 (42.8)	594 (555)	342 (62.2)	<0.001^*^
Relationship	1,370 (48.6)	685 (57.2)	477 (44.5)	208 (37.8)	
**Residency**					
Village	1,380 (49)	625 (52.2)	517 (43.2)	56 (4.7)	0.006^*^
City	1,292 (45.8)	512 (47.8)	508 (47.4)	51 (4.8)	
Refugee camp	147 (5.2)	243 (44.2)	267 (48.5)	40 (7.3)	
**Geographic area**					
West Bank	2,354 (83.5)	1,035 (86.4)	884 (82.5)	435 (79.1)	<0.001^*^
Gaza Strip	270 (9.6)	85 (7.1)	106 (9.9)	79 (14.4)	
Jerusalem	195 (6.9)	78 (6.5)	81 (7.6)	36 (6.5)	
**Educational level**					
Secondary or less	326 (11.6)	152 (12.7)	117 (10.9)	57 (10.4)	0.003^*^
College	2,211 (78.4)	900 (75.1)	870 (81.2)	441 (80.2)	
Master or doctorate	282 (10)	146 (12.2)	84 (7.8)	52 (9.5)	
**Health care worker**					
Yes	332 (11.8)	138 (11.5)	125 (11.7)	69 (12.5)	0.818
No	2,487 (88.2)	1,060 (88.5)	946 (88.3)	481 (87.5)	
**Monthly income**					
<2,000	568 (20.1)	198 (16.5)	231 (21.6)	139 (25.3)	<0.001^*^
2,000–5,000	1552 (55.1)	681 (56.8)	598 (55.8)	273 (49.6)	
>5,000	699 (24.8)	319 (26.6)	242 (22.6)	138(25.1)	
**Smoking/Shisha**					
Yes	693 (24.6)	289 (24.1)	262 (24.5)	142 (25.8)	0.742
No	2,126 (75.4)	909 (75.9)	809 (75.5)	408 (74.2)	
**High risk group in home**					
Yes	1,283 (45.5)	513 (42.8)	484 (45.2)	286 (52)	0.002^*^
No	1,536 (54.5)	685 (57.2)	587 (54.8)	264 (48)	

* *It means that the p-value is statistically significant*.

Most of the participants (78.4%) were currently studying in college or have graduated recently. On the other hand, 10% of them are studying a master or doctorate degree. Almost one quarter (24.6%) were smokers, and only 11.8% were health-care workers. About 45.5% reported that they had a high-risk individual living with them currently.

Results showed that 1,144 (40.6%), 1,261 (44.7%), and 1,283 (45.5%) of respondents had low levels of stay-at-home commitment, commitment to inside home precautions, and understanding of quarantine, respectively.

### Quarantine Characteristics of the Population

As shown in Table 3, 98% of respondents believed that quarantine is important, and 2,173 (77.1%) expressed that they afraid of getting the COVID-19 or transmitting it to others. Only 14.9% of respondents had jobs that required them to go outdoors, and only 85 (3%) had at least one of their relatives with confirmed COVID-19. The two most common sources of information about quarantine and precautions were social media and television or radio (59.5 and 18.6%, respectively). Nearly 80.2% admitted that they are properly informed about the quarantine. In addition, 29.3% reported that they had inadequate food supply to withstand the quarantine period.

**Table 3:** Bivariate analysis of quarantine characteristics with depression severity (*P*-value presented was Chi-square significance; N=2819).

		**Depression severity**			
**Variables**	**N (%)**	**Normal*n* = 1,198**	**Mild to moderate *n* = 1071**	**Severe to extremely sever*n* = 550**	***P-*value**
**Do you think quarantine is important?**					
Yes	2,763 (98)	1,175 (98.1)	1,055 (98.5)	533 (96.9)	0.090
No	56 (2)	23 (1.9)	16 (1.5)	17 (3.1)	
**Type of quarantine**					
Obliged to stay at home	2,398 (85.1)	1,006 (84)	924 (86.3)	468 (85.1)	0.308
I have to work outside home	421 (14.9)	192 (16)	147 (13.7)	82 (14.9)	
**Any of relatives or acquainted infected?**					
Yes	85 (3)	23 (1.9)	38 (3.5)	24 (4.4)	0.009^*^
No	2,734 (97)	1,175 (98.1)	1,033 (96.5)	526 (95.6)	
**Afraid of getting COVID-19 or transmit it?**					
Yes	2,173 (77.1)	883 (73.7)	865 (80.8)	425 (77.3)	<0.001^*^
No	646 (22.9)	315 (26.3)	206 (19.2)	125 (22.7)	
**Properly informed about quarantine**					
Yes	2,262 (80.2)	1,002 (83.6)	858 (80.1)	402 (73.1)	<0.001^*^
No	557 (19.8)	196 (16.4)	213 (19.9)	148 (26.9)	
**Source of information**					
Television or radio	525 (18.6)	24 (20.6)	186 (17.4)	92 (16.7)	0.003^*^
Official government agencies	359 (12.7)	177 (14.8)	127 (11.9)	55 (10)	
A health care worker	159 (5.6)	74 (6.2)	55 (5.1)	30 (5.5)	
Social media	1,676 (59.5)	668 (55.8)	659 (61.5)	349 (63.5)	
Conversation with other people	100 (3.6)	32 (2.7)	44 (4.1)	24 (4.4)	
**Enough food supply to withstand quarantine period?**					
Yes	1,994 (70.7)	891 (74.4)	782 (71.7)	341 (62)	<0.001^*^
No	825 (29.3)	307 (25.6)	309 (28.9)	209 (38)	
**Quarantine duration**					
1–2 weeks	187 (6.6)	80 (6.7)	74 (6.9)	33 (6)	0.280
2–3 weeks	847 (30.1)	390 (32.6)	301 (28.1)	156 (28.4)	
3–4 weeks	786 (27.9)	327 (27.3)	300 (28)	159 (28.9)	
>4 weeks	999 (35.4)	401 (33.5)	396 (37)	202 (36.7)	
**Average hours out home before quarantine**					
<2 h	584 (20.7)	251 (21)	209 (19.5)	124 (22.5)	0.020^*^
2–6 h	776 (27.5)	314 (26.2)	335 (31.3)	127 (23.1)	
6–10 h	1,075 (38.2)	470 (39.2)	391 (36.5)	214 (38.9)	
>10 h	384 (13.6)	163 (13.6)	136 (12.7)	85 (15.5)	
**Stay at home commitment**					
Low level	1,144 (40.6)	501 (41.8)	424 (39.6)	219 (39.8)	0.514
High level	1,675 (59.4)	697 (58.2)	647 (60.4)	331 (60.2)	
**Commitment to inside home precautions**					
Low level	1,261 (44.7)	503 (42)	506 (47.2)	252 (45.8)	0.036^*^
High level	1,558 (55.3)	695 (58)	565 (52.8)	298 (54.2)	
**Understanding of quarantine**					
Low level	1,283 (45.5)	534 (44.6)	487 (45.5)	262 (47.6)	0.490
High level	1,536 (54.5)	664 (55.4)	584 (54.5)	288 (52.4)	
Self-rating of quarantine commitment	2,819 (100)	Mean = 8.43 SD = 1.9	Mean = 8.55 SD = 1.7	Mean = 8.54 SD =1.9	0.230

* *It means that the p-value is statistically significant*.

Quarantine duration ranged from <2 weeks at 6.6% to more than 4 weeks at 35.4%. Nearly 38.2% used to spend between 6 and 10 h outside the home before quarantine, 20.7% spent <2 h, and only 13.6% spent more than 10 h (see Table 3 for more details).

### Prevalence of Depression in Bivariate Analysis

The prevalence of depression was 57.5% (*n* = 1,621; 38% with mild/moderate depression and 19.5% with severe/extremely severe).

**Figure 1:**
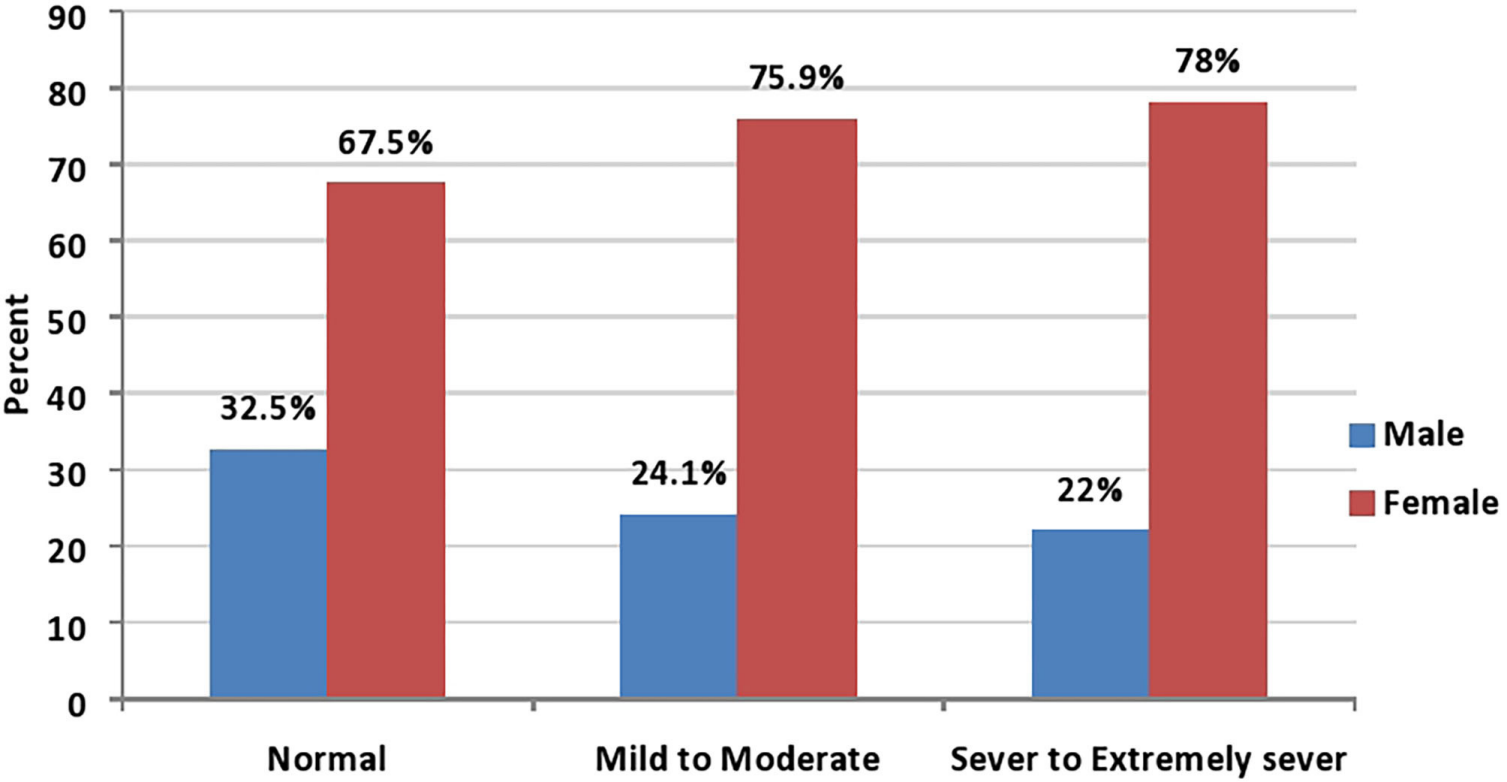
Sex distribution among depression severity (*P*-value < 0.001; *N* = 2,819).

**Figure 2:**
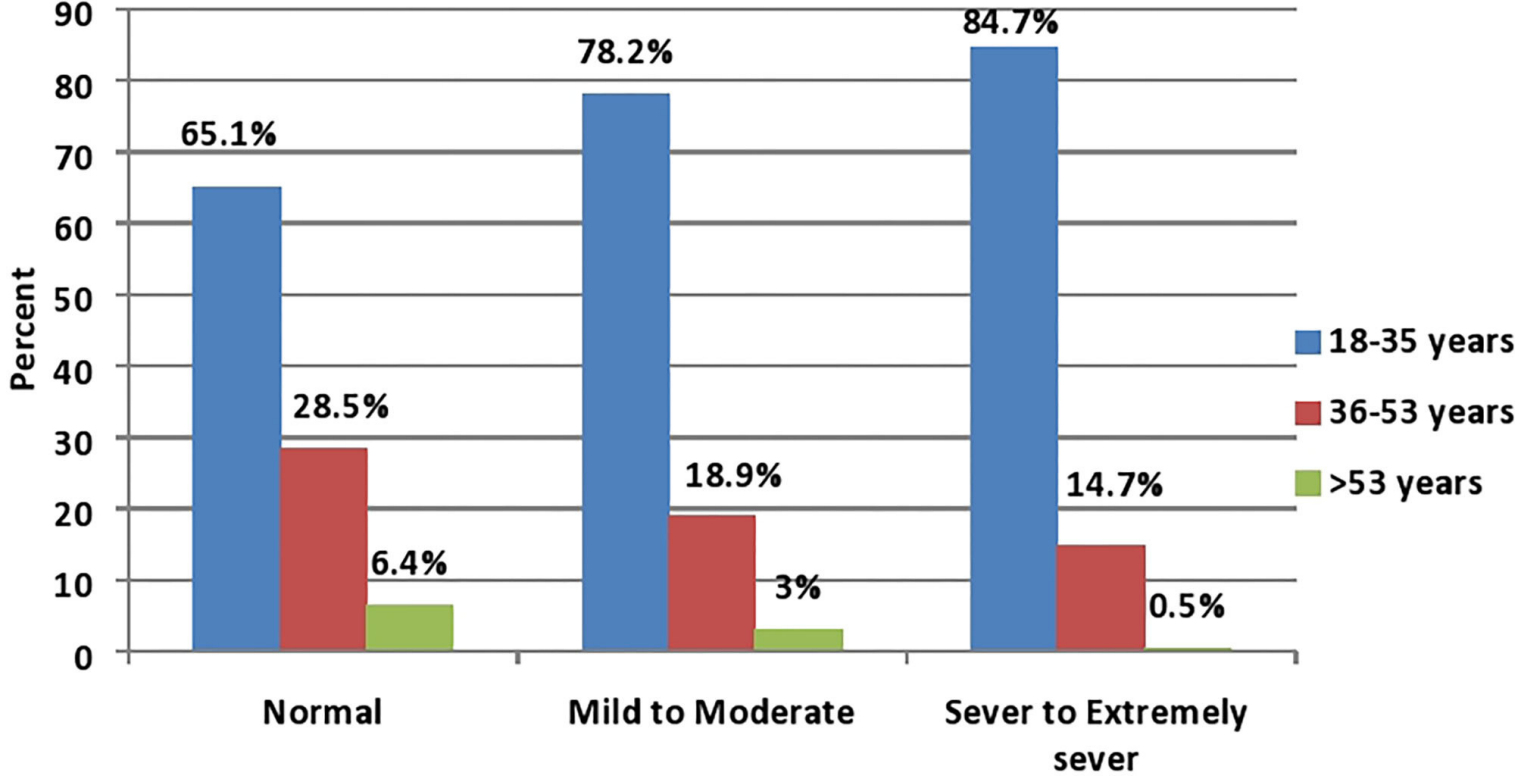
Age distribution among depression severity (*P*-value < 0.001; *N* = 2,819).

In the bivariate analysis, statistically significant associations were found between depression severity and each of age, sex, social status, residency, geographic area, educational level, monthly income, smoking, and the presence of a high-risk individual (*P*-value <0.05; see Table 2). Females represented the majority in all depression degrees as compared with males with 67.5, 75.9, and 78% in normal, mild/moderate, and severe/extremely severe degrees, respectively (Figure 1; *P*-value <0.001). Similarly, the young age group (18–35 years) represented the majority in all depression degrees with 65.1, 78.2, and 84.7% in normal, mild/moderate, and severe/extremely severe degrees, respectively (Figure 2; *P*-value <0.001).

Nearly 48.5% of the total refugee camps residents had mild/moderate depression, and 7.3% had severe/extremely severe degree of depression; and this represents the highest percentage distribution as compared with city and village residents among depression severity degrees.

West Bank residents represented the majority among all depression degrees as compared with Gaza and Jerusalem (86.4% of the normal, 82.5% of the mild/moderate, and 79.1% of the severe/extremely severe degrees). Statistically significant associations were found between depression severity and having enough food supply to withstand quarantine, average hours out of the home before quarantine, and commitment to inside home precautions (*P*-value < 0.05; see Table 3).

### Multinomial Regression Analysis of Depression Severity Predictors

Multinomial regression model for the variables associated with depression severity is shown in Table 4. As shown, depression severity was negatively associated with age {mild/moderate degree [OR (95% CI) = 0.98 (0.97–0.99)]} and {severe/extremely severe [OR (95% CI) = 0.96 (0.94–0.97)]} as compared with normal degree. Males were significantly less likely to have a higher depression degree as compared with females {mild/moderate degree [OR (95%CI) = 0.69 (0.57–0.85)]} and {severe/extremely severe [OR (95%CI) = 0.52 (0.40–0.86)]}. However, those who reported inadequate food supply were associated with a higher degree of depression compared with normal degree. Single persons were significantly more likely to have mild/moderate depression compared with those in a relationship [OR (95% CI) = 1.31 (1.05–1.64)].

**Table 4:** Multinomial regression model for the variables associated with depression severity^#^ (*N* = 2,819).

	**Mild to moderate**				**Severe to extremely sever**			
**Variable**	**B**	**SE**	**OR (95%CI)**	***P*-value**	**B**	**SE**	**OR (95%CI)**	***P*-value**
Age (continuous)	−0.02	0.005	0.98 (0.97–0.99)	0.001	−0.04	0.008	0.96 (0.94–0.97)	<0.001
**Sex**								
Male	−0.37	0.104	0.69 (0.57–0.85)	<0.001	−0.65	0.134	0.52 (0.40–0.68)	<0.001
Female^*^	–	–	–	–	–	–	–	–
**Social status**								
Single	0.27	0.114	1.31 (1.05–1.64)	0.017	0.25	0.143	1.29 (0.97–1.71)	0.076
In relationship^*^	–	–	–	–	–	–	–	–
**Residency**								
Village	0.002	0.217	1.00 (0.66–1.53)	0.993	−0.34	0.243	0.71 (0.44–1.15)	0.162
City	0.19	0.213	1.21 (0.80–1.84)	0.372	−0.11	0.237	0.89 (0.56–1.42)	0.631
Refugee camp^*^	–	–	–	–	–	–	–	–
**Geographic area**								
West Bank	−0.15	0.174	0.86 (0.61–1.21)	0.394	0.11	0.224	1.11 (0.72–1.73)	0.631
Gaza Strip	−0.18	0.234	0.83 (0.53–1.32)	0.432	0.35	0.283	1.42 (0.82–2.48)	0.212
Jerusalem^*^	–	–	–	–	–	–	–	–
**Educational level**								
Secondary or less	0.07	0.198	1.07 (0.73–1.57)	0.739	−0.35	0.246	0.71 (0.44–1.15)	0.161
College	0.19	0.155	1.20 (0.89–1.63)	0.231	−0.23	0.189	0.80 (0.55–1.16)	0.231
Master or doctorate^*^	–	–	–	–	–	–	–	–
**Monthly income**								
<2,000	0.39	0.145	1.48 (1.11–1.97)	0.007	0.17	0.173	1.18 (0.84–1.66)	0.335
2,000–5,000	0.18	0.109	1.20 (0.97–1.48)	0.069	−0.12	0.135	0.89 (0.69–1.16)	0.394
>5,000^*^	–	–	–	–	–	–	–	–
**High risk group in home**								
No	−0.10	0.088	0.91 (0.76–1.08)	0.271	−0.38	0.109	0.69 (0.56–0.85)	0.001
Yes^*^	–	–	–	–	–	–	–	–
**Any of relatives or acquainted infected?**								
No	−0.66	0.275	0.52 (0.30–0.89)	0.016	−0.83	0.310	0.44 (0.24–0.80)	0.007
Yes^*^	–	–	–	–	–	–	–	–
**Afraid of getting COVID-19 or transmit it?**								
No	−0.44	0.106	0.65 (0.53–0.80)	<0.001	−0.25	0.129	0.78 (0.61–1.01)	0.057
Yes^*^	–	–	–	–	–	–	–	–
**Properly informed about quarantine**								
No	0.15	0.115	1.17 (0.93–1.46)	0.187	0.44	0.133	1.56 (1.20–2.02)	0.001
Yes^*^	–	–	–	–	–	–	–	–
**Source of information**								
Television or radio	−0.40	0.261	0.67 (0.40–1.12)	0.127	−0.23	0.312	0.80 (0.43–1.47)	0.470
Official government agencies	−0.53	0.269	0.59 (0.35–1.00)	0.050	−0.56	0.327	0.57 (0.30–1.08)	0.085
A health care worker	−0.58	0.301	0.56 (0.31–1.01)	0.055	−0.52	0.363	0.59 (0.29–1.21)	0.151
Social media	−0.28	0.246	0.76 (0.47–1.23)	0.262	−0.15	0.291	0.86 (0.49–1.52)	0.602
Conversation with other people^*^	–	–	–	–	–	–	–	–
**Enough food supply to withstand quarantine period?**								
No	0.20	0.103	1.22 (1.00–1.49)	0.056	0.62	0.122	1.86 (1.47–2.37)	<0.001
Yes^*^	–	–	–	–	–	–	–	–
**Average hours out home before quarantine**								
<2 h	−0.16	0.159	0.85 (0.63–1.17)	0.321	−0.27	0.188	0.77 (0.53–1.11)	0.156
2–6 h	0.04	0.150	1.04 (0.78–1.40)	0.779	−0.53	0.186	0.59 (0.41–0.85)	0.004
6–10 h	−0.10	0.142	0.91 (0.69–1.20)	0.481	−0.28	0.169	0.76 (0.55–1.06)	0.103
>10 h^*^	–	–	–	–	–	–	–	–
**Commitment inside home precautions**								
Low level	0.18	0.089	1.20 (1.01–1.43)	0.040	0.09	0.110	1.09 (0.88–1.35)	0.44
High level^*^	–	–	–	–	–	–	–	–

# *Reference category: Normal*;

* *Reference category. OR, Odds ratio; CI, Confidence interval*.

*Likelihood Ratio Test of the final model fitting significance was <0.001. Pearson chi-square test for model goodness-of-fit was significance 0.336*.

Those with a monthly income of <2,000 New Israeli Shekels (<555 USD) were significantly more likely to have mild/moderate depression than those with normal degree and higher monthly incomes [OR (95% CI) = 1.48 (1.11–1.97)]. Those who do not have a high-risk individual living with them were significantly less likely to have severe/extremely severe depression than normal degree [OR (95% CI) = 0.69 (0.56–0.85)]. Moreover, those who reported that none of their relatives were infected with the COVID-19 and those who are not afraid of being infected were inversely related to a higher degree of depression severity (see Table 4).

Being not properly informed about quarantine was a significant positive predictor of severe/extremely severe depression compared with normal degree [OR (95% CI) = 1.56 (1.20–2.02)]. On the other hand, those who usually spend lesser hours (2–6 h) out the home before quarantine were significantly less likely to have severe/extremely severe depression compared with more hours (>10 h) and normal degree [OR (95% CI) = 0.59 (0.41–0.85)]. However, those who reported a low level of commitment to inside home precautions were more likely to have mild/moderate depression [OR (95% CI) = 1.20 (1.01–1.43)].

It should be noted that residency, geographic area, educational level, and source of information did not remain significant after adjusting for other variables in the multinomial regression model.

## Discussion

In this study, the prevalence of depression among the Palestinian community during the COVID-19 pandemic was found to be 57.5% (38% with mild/moderate depression and 19.5% with severe/extremely severe). A cross-sectional general population study of 916 Palestinian adults in 2007 reported the 1-month prevalence of major depressive episodes was 10.6% (10). Our study reported a five-fold increase in the prevalence of depression during the COVID-19 in Palestine. This finding represents a high degree of depression at the general population level in Palestine. Moreover, a recent study in Italy reported a prevalence of 17.3% of depression among the Italian general population during the COVID-19 pandemic (11). In Spain (12), a similar study evaluated the prevalence of depression among the Spanish general population and found that depression was around 19%. Whereas in the UK, the prevalence was 22.12% during the COVID-19 pandemic among the general population (13).

A possible explanation for our finding of a high level of depression could be that in Italy and Spain the prevalence was evaluated in the early stages of the COVID-19 pandemic, while in this study, it has been evaluated at the late stage of the pandemic. Moreover, Palestine is classified as a low- to middle- income country (14) and has a low socioeconomic status with a special geopolitical situation, which could add to the negative mental health effects during the COVID-19 in Palestine. Another study in China (5) reported a prevalence of depression during the COVID-19 for inpatient health questionnaire to be around 50.7%, which is near our findings. This raises an alarming sign in Palestine, as the participants were not COVID-19 patients, and the result is higher than that reported among patients in other countries. We found no study from the Arab world that evaluated depression during the COVID-19 for comparison, and this study could be a starting point for other future studies to cite from the Arab world.

Another important finding in this study was that females were associated with mild/moderate degree and severe/extremely severe degree of depression as compared with males. This is consistent with two studies during the COVID-19 lockdown measures that showed that females were more vulnerable to negative emotional outcomes such as depression (11, 15). The cultural background and the fact that home responsibility relied mainly on females in Palestine could add to more emotional and depressive effects among females during the lockdown measures. Moreover, females are a vulnerable group in the Palestinian community. Furthermore, females are the main component of the young age adult group in this study, and this might partially contribute to the high prevalence of depression among this gender–age group. Interestingly enough, age showed an inverse relationship with depression severity during the lockdown and massive quarantine in Palestine during the COVID-19. Our results are in accordance with those of similar studies in Italy and Spain conducted during the same pandemic (11, 12). It is noteworthy that young adults in the Palestinian community represent university students mainly, and as a result of lockdown, online educational activities were implemented to continue the ongoing academic semester. This group of young adults had to deal with the dramatic changes in requirements to pass the academic semester. At the same time, this group of the young population includes newly graduated adults who are still unemployed or newly employed, so they tend to have less sustained monthly income than older adults. This finding should be taken seriously by decision makers since the young age group is the main productive group in Palestine and worldwide. Therefore, losing productivity might have impacts not only at the personal level but also at the national socioeconomic level of the country.

Those who do not have a high-risk individual inside the home were less likely to have severe/extremely severe depression. This result was also found in the UK (13), which demonstrated that people who have a relative or family member with a preexisting medical condition were more likely to have depressive symptoms.

Monthly income was inversely related to the degree of depression. This is consistent with a study conducted in Southwestern China (4) that reported that a high average household income group had a significantly lower level of depression than a low average household income group due to the economic impact of the COVID-19 and failure to cope with financial problems. In another study in the UK (13), a significant association was also found between loss of income and depression. However, those who reported having inadequate food supply were more likely to have a higher degree of depression compared with normal degree. This may be because those people who reported a shortage of food supply were potentially from low-income group or they lost their jobs as a consequence of lockdown measures. Thus, they had been exposed to a difficult situation without financial support.

Single persons were more likely to have mild/moderate depression than those in a relationship. These findings are congruent with other results that come from China (4). Single persons could be away from their beloved ones, and this might contribute to more depressed emotions among the general population in Palestine. Moreover, those who reported that none of their relatives were infected with the COVID-19 and those who were not afraid of being infected were less likely to have a higher degree of depression severity. All these factors could stimulate another psychological burden on people, which may explain why they showed a significant association with the higher degree of depression. Furthermore, those who usually spend lesser hours (2–6 h) out the home before quarantine were less likely to have severe/extremely severe depression than those who spend more hours (>10 h) and normal degree. This could be because those who used to go outside the home more frequently tend to find more difficulty engaging in inside home activities than persons who usually spend less time outside home.

It is worth mentioning that in our study educational level had no impact on depression severity. This contradicts other similar studies that found a significant association between educational level and depression degree (4, 11, 15). In our study, this could be because those who have a secondary level of education or less were not engaged in online learning, and they are dependent on their parents in the Palestinian community. However, those with a college degree and higher might have higher socioeconomic status and a stable monthly income.

It should be noted that the depression severity may be also attributed to the unclear future and whether or not the adopted governmental plan will overcome the build-up of troubles and consequences of the extreme lockdown. However, we did not investigate these factors in this study, and therefore, further future studies on this perspective might be needed.

Our study could be limited by the sampling technique, and therefore, selection bias might be encountered. For example, it was noticed that 72.8% of the sample were female which might overestimate the depression severity and therefore, our depression rates should be interpreted with caution. Furthermore, due to social distancing during quarantine, we disseminated the survey on social media, and this might in part exclude people who did not have access to the internet and social media. On the other side, this was the only possible procedure during the lockdown measures, and it was useful in collecting the required information as fast and as safely as possible. This study was a cross-sectional web-based survey, and therefore, recall and/or systematic biases might have been occurred where overestimation or underestimation of some measures might have been occurred due to self-reporting. It should be noted however that this study has several strengths, including a large sample size and the sampling timeframe that corresponded to the peak surge of the COVID-19 cases in Palestine, which had 613 cases and 5 death when this paper was being written (16). Taking into account the worldwide nature of the risk in this pandemic, we strongly believe that these data could provide important and useful information to be generalized to other countries and to future pandemics.

## Conclusions

High rates of depression and various predictors of its severity among the Palestinian community during lockdown periods of the COVID-19 pandemic were reported. The high prevalence of depression (57.5%) forces the authorities and decision makers to immediately intervene to address the effects of this concerning rise among Palestinians along with the public health measures taking place. Strategic long-term policy to address the pandemic ramifications, including depression, by implementing comprehensive interventions taking into account the socioeconomic disparities, vulnerability, and inequities are crucial to emerge from this crisis in Palestine mainly among the young age group and female gender, who showed more vulnerability to depression.

We strongly believe that this study could help in generating socioeconomic and health initiatives to prevent and manage the pandemic's depression severity. It is crucial for communities to move forward and emerge from the crisis impacts. Furthermore, it is essential to provide psychological counseling and treatment during and after pandemic periods for these groups in Palestine. A post-pandemic assessment of depression among the Palestinian community is recommended to highlight any improvement or deterioration.

## Data Availability Statement

The original contributions presented in the study are included in the article/supplementary materials, further inquiries can be directed to the corresponding author/s.

## Ethics Statement

The studies involving human participants were reviewed and approved by IRB of An-Najah National University. All respondents provided online informed consent before starting the questionnaire. The IRB approved our request for a waiver of documentation of this method of obtaining consent.

## Author Contributions

HA, TA, NY, and MH-Y designed study protocol and drafted the manuscript. HA coordinated the study protocol and conducted the statistical analysis. TA, NY, and MH-Y collected the data. All authors contributed to the article and approved the submitted version.

## Conflict of Interest

The authors declare that the research was conducted in the absence of any commercial or financial relationships that could be construed as a potential conflict of interest.
